# LightLogR: Reproducible analysis of personal light exposure data

**DOI:** 10.21105/joss.07601

**Published:** 2025-03-13

**Authors:** Johannes Zauner, Steffen Hartmeyer, Manuel Spitschan

**Affiliations:** 1 Technical University of Munich, TUM School of Medicine and Health, Department Health and Sport Sciences, Chronobiology & Health, Munich, Germany; 2 Max Planck Institute for Biological Cybernetics, Max Planck Research Group Translational Sensory & Circadian Neuroscience, Tübingen, Germany; 3 École Polytechnique Fédérale de Lausanne (EPFL), School of Architecture, Civil and Environmental Engineering (ENAC), Laboratory of Integrated Performance in Design (LIPID), Lausanne, Switzerland; 4 TUM Institute for Advanced Study (TUM-IAS), Technical University of Munich, Garching, Germany; 5 TUMCREATE Ltd., Singapore, Singapore

## Abstract

Light plays an important role in human health and well-being, which necessitates the study of the effects of personal light exposure in real-world settings, measured by means of wearable devices. A growing number of studies incorporate these kinds of data to assess associations between light and health outcomes. Yet with few or missing standards, guidelines, and frameworks, it is challenging setting up measurements, analysing the data, and comparing outcomes between studies. Overall, time series data from wearable light loggers are significantly more complex compared to controlled stimuli used in laboratory studies. In this paper, we introduce LightLogR, a novel resource to facilitate these research efforts. The package for R statistical software is open-source and permissively MIT-licenced. As part of a developing software ecosystem, LightLogR is built with common challenges of current and future datasets in mind. The package standardises many tasks for importing and processing personal light exposure data. It allows for quick as well as detailed insights into the datasets through summary and visualisation tools. Furthermore, LightLogR incorporates major metrics commonly used in the field (61 metrics across 17 metric families), all while embracing an inherently hierarchical, participant-based data structure.

## Statement of need

Personalised luminous exposure data are progressively gaining importance across various domains, including research, occupational affairs, and lifestyle tracking. Data are collected through a increasing selection of wearable light loggers and dosimeters, varying in size, shape, functionality, and output format (Hartmeyer et al., 2023). Despite or potentially because of numerous use cases, the field still lacks a unified framework for collecting, validating, and analyzing the accumulated data (Hartmeyer et al., 2023; Spitschan et al., 2022). This issue increases the time and expertise necessary to handle such data and also compromises the FAIRness (findability, accessibility, interoperability, reusability) (Wilkinson et al., 2016) of the results, especially for meta-analyses (Vries et al., 2024).

**Figure 1:**
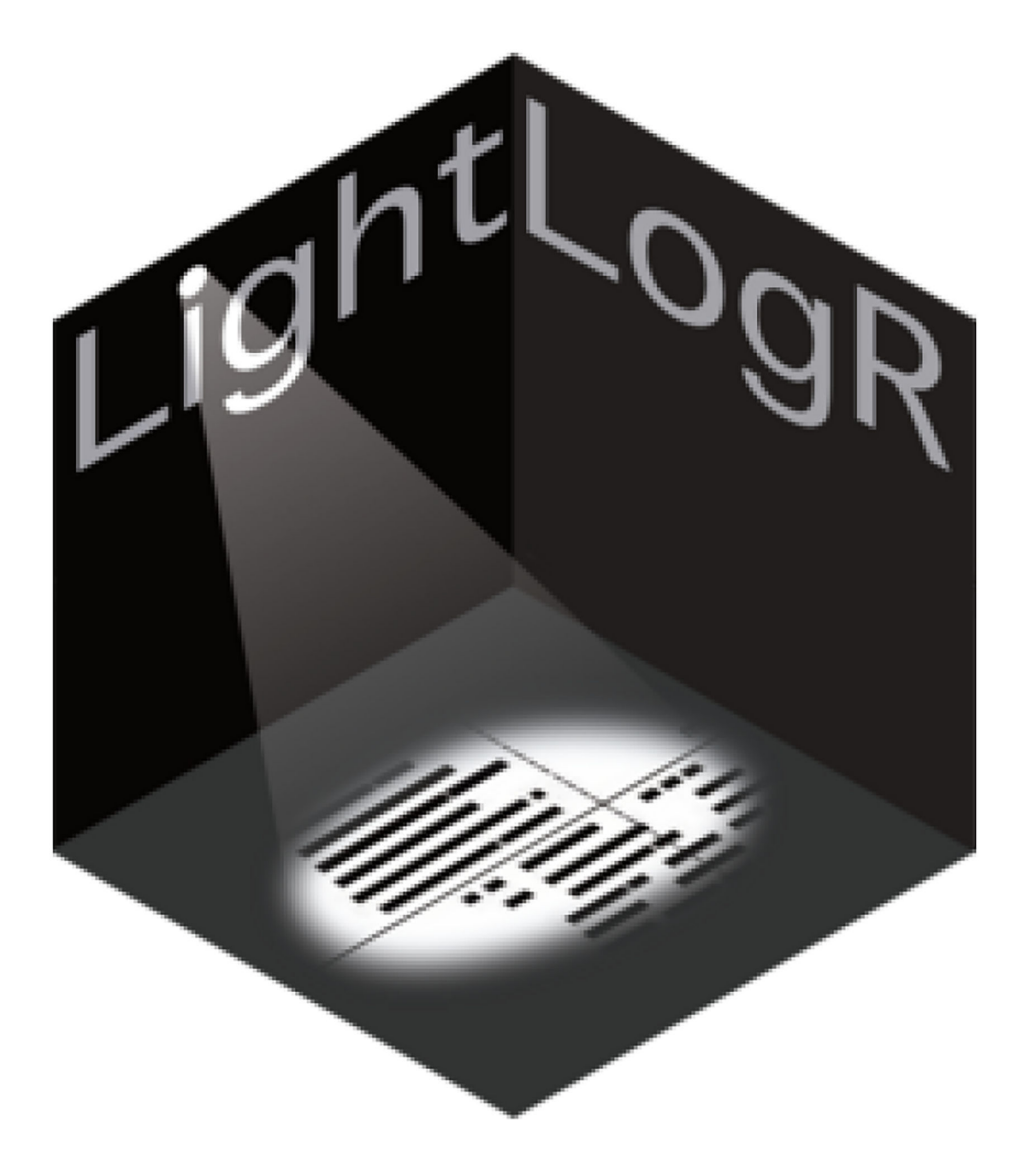
LightLogR logo

LightLogR was designed to be used by researchers who deal with personal light exposure data collected from wearable devices (Figure 1). These data are of interest for various disciplines, including chronobiology, sleep research, vision science and epidemiology, as well as for post-occupancy evaluations in architecture and lighting design. The package is intended to streamline the process of importing, processing, and analysing these data in a reproducible and transparent manner. The package is available on GitHub (Zauner et al. (2025b)) and CRAN (Zauner, Hartmeyer, et al. (2024)), has a dedicated website for documentation and tutorials (Zauner et al. (2025a)), and releases are archived on Zenodo (Zauner et al. (2025c)).

**Figure 2:**
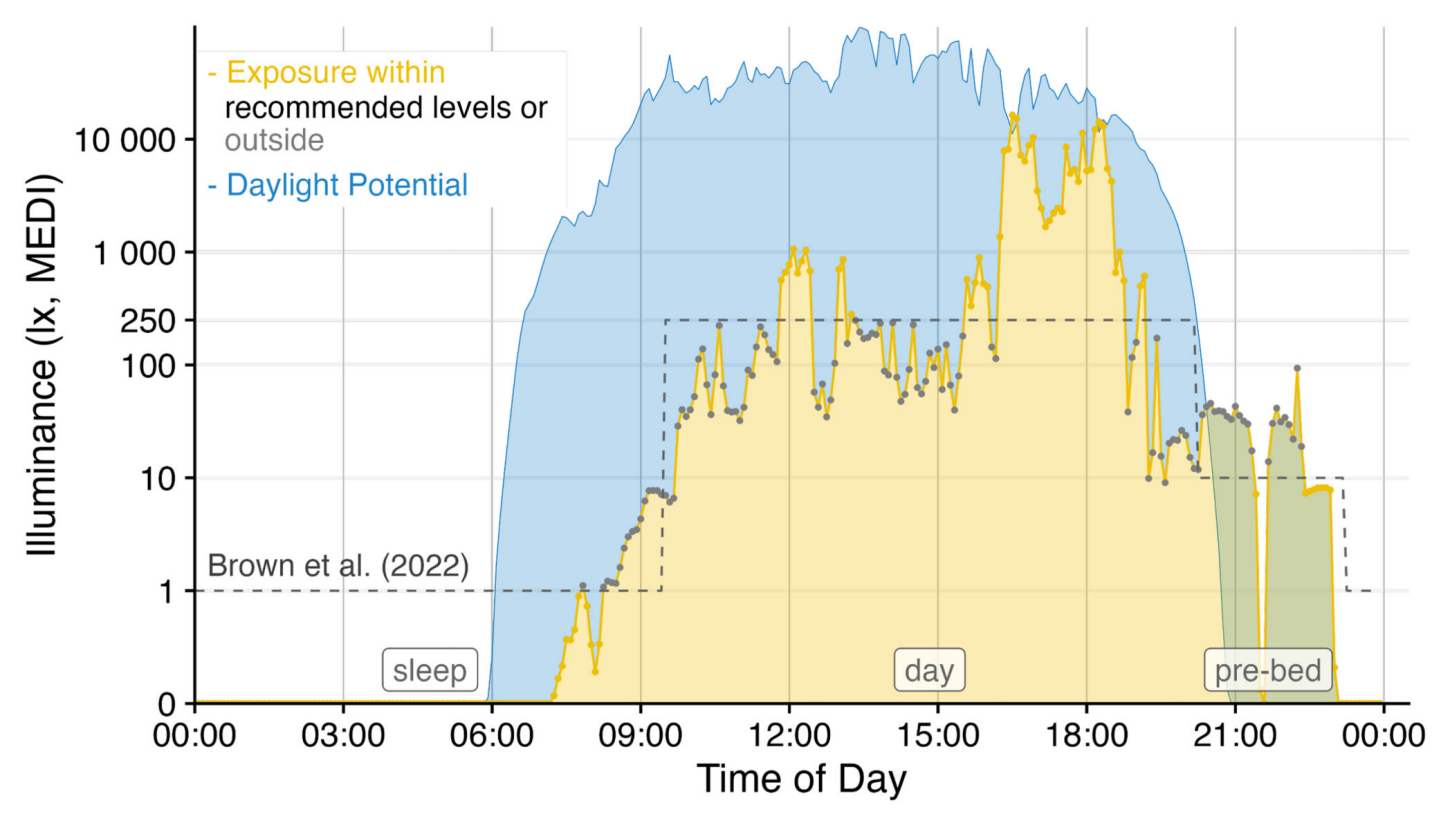
Light logger data can powerfully convey insights into personal light exposure and health-related outcomes. LightLogR facilitates the import and combination of different data sources into a coherent data structure, as seen here by combining environmental daylight availability and personal light exposure with data from a sleep diary. The visualisation functions in the package further allow customisation to produce publication-ready figures. This figure was created with the ‘gg_day()’ function. The creation process is part of a tutorial (Zauner et al., 2023) on several key functions in the package.

LightLogR’s key features include:

▪ a growing list of supported devices with pre-defined import functions tailored to their data structure (17 at the time of writing, see Table 1),
▪ preprocessing functions to combine different time series, aggregate and filter data, and find and deal with implicitly missing data,
▪ visualisation functions to quickly explore the data. These functions are based on the popular ggplot2 (Wickham, 2016) plotting package and are designed to be easily customisable to construct publication-ready figures (see, Figure 2),
▪ a large and growing set of metrics that cover most if not all major approaches found in the literature (at the time of writing 61 metrics across 17 metric families, see Table 2) and (Hartmeyer et al., 2023)), accessible via a consistent function interface.

LightLogR is already being used in several research projects across scientific domains, including:

▪ an ongoing cohort study to collect light exposure data across different geolocations (Guidolin et al., 2024),
▪ an ongoing cohort study to collect year-long datasets of various types of environmental and behavioural data (Biller et al., 2024),
▪ a novel power analysis method for personal light exposure data (Zauner, Udovicic, et al., 2024),
▪ an intervention study on the effects of light on bipolar disorder (Roguski et al., 2024),
▪ an intervention study on exposure to bright light during afternoon to early evening on later evening melatonin release in adolescents (Lazar et al., 2024),
▪ an observational study on the wearing compliance of personal light exposure (Stefani et al., 2024),
▪ an observational study on the differences in light exposure and light exposure related behaviour between Malaysia and Switzerland (Biller et al., 2025),
▪ an intervention study on sex and seasonal changes in human melatonin suppression and alerting response to moderate light (Fazlali et al., 2024),
▪ an observational study on light exposure, sleep, and circadian rhythms in hospital shift workers (publication in progress).

## Figures and Tables

**Table 1:** Devices supported for import in version 0.5.0

Device Name	Manufacturer
Actiwatch Spectrum	Philips Respironics
ActLumus	Condor Instruments
ActTrust	Condor Instruments
DeLux	Intelligent Automation Inc.
GENEActiv^1^	Activeinsights
Kronowise	Kronohealth
Lido	Lucerne University of Applied Sciences and Arts
LightWatcher	Object-Tracker
LIMO	École nationale des travaux publics de l’État (ENTPE)
LYS Button	LYS Technologies
Motion Watch 8	CamNtech
melanopiQ Circadian Eye	Max Planck Institute for Biological Cybernetics
XL-500 BLE	NanoLambda
OcuWEAR	Ocutune
Speccy	Monash University Malaysia
SpectraWear	University of Manchester
VEET	Meta Reality Labs

1 Available after processing of the data using GGIR (Migueles et al., 2019).

**Table 2:** metrics available in version 0.5.0

Metric Family	Submetrics	Note	Documentation
Barroso	7		barroso_lighting_metrics()
Bright-dark period	4x2	bright / dark	bright_dark_period()
Centroid of light exposure	1		centroidLE()
Disparity index	1		disparity_index()
Duration above threshold	3	above, below, within	duration_above_threshold()
Exponential moving average (EMA)	1		exponential_moving_average()
Frequency crossing threshold	1		frequency_crossing_threshold()
Intradaily Variance (IV)	1		intradaily_variability()
Interdaily Stability (IS)	1		interdaily_stability()
Midpoint CE (Cumulative Exposure)	1		midpointCE()
nvRC (non-visual circadian response)	4		nvRC(), nvRC_circadianDisturbance(), nvRC_circadianBias(), nvRC_relativeAmplitudeError()
	nvRD (non-visual direct response)	2		nvRD(), nvRD_cumulative_response()
	Period above threshold	3	above, below, within	period_above_threshold()
	Pulses above threshold	7x3	above, below, within	pulses_above_threshold()
Threshold for duration	2	above, below	threshold_for_duration()
Timing above threshold (TAT)	3	above, below, within	timing_above_threshold()
**Total:** **17 families**	**61 metrics**		
